# SSR-based genetic diversity and population structure analysis of 144 core pea (*Pisum sativum* L.) accessions

**DOI:** 10.3389/fpls.2026.1806277

**Published:** 2026-06-30

**Authors:** Jiu-Yan Zhao, Feng-Jing Song, Jun-Jie Hao, Shi-Zuo Qiu, Xiao Cui, Gong-Qiang Jiao, Huan Song, Jing Bai, Guan Li, Peng Chen, Na-Na Li, Xiao-Yan Zhang

**Affiliations:** 1 Qingdao Academy of Agricultural Sciences, Qingdao, China; 2 College of Agronomy, State Key Laboratory of Crop Biology, Shandong Agricultural University, Taian, China; 3 Mid-Term National Germplasm Resource Bank for Drought-Resistant and Salt-Alkali Tolerant Crops (Jinan); National Salt-Alkali Tolerant Crop Germplasm Resources Nursery (Dongying); Institute of Crop Germplasm Resources, Shandong Academy of Agricultural Sciences, Jinan, China; 4 Department of Rehabilitation Medicine, The Second Affiliated Hospital of Shandong First Medical University, Taian, China; 5 Shandong Yuwang Ecological Food Industry Co., Ltd., Dezhou, China

**Keywords:** AMOVA, genetic diversity, germplasm resources, *Pisum sativum* L., population structure, SSR markers

## Abstract

Pea (*Pisum sativum* L.), a widely cultivated cool-season legume crop globally, serves as a high-quality source of plant protein for humans. Despite its critical importance, systematic evaluations of the genetic diversity and population structure of a phenotype−guided core collection integrating germplasm from multiple regions remain limited. In this study, a core collection of 144 pea accessions with important breeding value was evaluated for genetic diversity using Simple Sequence Repeat (SSR) molecular markers to elucidate their population genetic structure. The genetic diversity results revealed an average number of alleles (Na) of 4.923, an average number of effective alleles (Ne) of 2.130, an average expected heterozygosity (He) of 0.468, and an average observed heterozygosity (Ho) of 0.207. The average Shannon’s information index (I) and average polymorphism information content (PIC) were 0.895 and 0.4219, respectively, indicating relatively rich genetic diversity within the pea population. Population structure analysis (K = 2) divided the accessions into two major genetic groups, with 32 accessions assigned to Group I, 61 to Group II, and 51 accessions (35.4%) showing admixed ancestry. The UPGMA dendrogram and principal component analysis were broadly consistent with this grouping. Analysis of molecular variance (AMOVA) revealed that most genetic variation was distributed within populations (87%), whereas variation among populations accounted for 13% (Fst = 0.129, P < 0.001). These findings provide a genetic stratification framework that can guide germplasm conservation, parental selection, and future marker−assisted breeding efforts in pea.

## 1 Introduction

Pea (*Pisum sativum* L.) is an important cool-season legume crop that plays a critical role in global food security and sustainable agricultural systems (Graham and Vance, 2003; Smýkal et al., 2015). Not only is it a high-quality source of plant protein, but it also enhances soil fertility through symbiotic nitrogen fixation with rhizobia, making it a key component of environmentally friendly crop rotation systems (Bohlool et al., 1992; Dahl et al., 2012). However, current pea breeding and production face numerous challenges, such as stagnating yield growth and insufficient resistance to biotic and abiotic stresses. These bottlenecks are often attributed to limited genetic gains resulting from the narrow genetic base of modern cultivated varieties (Rubiales et al., 2015; Swarup et al., 2021). Therefore, systematically exploring, evaluating, and utilizing the rich genetic variation present in existing germplasm resources is a crucial prerequisite for broadening the genetic base and achieving breakthroughs in breeding.

Simple sequence repeat (SSR) markers have been widely used for germplasm characterization, population structure analysis, and parental selection in various crops due to their high polymorphism, codominant inheritance, good reproducibility, and low cost per sample. For example, ISSR markers have been successfully employed to evaluate genetic diversity and population structure in peach (*Prunus persica* L.) germplasm (Demirel et al., 2024), while SSR markers have been used to investigate genetic relationships and gene flow in orchid species (Palaz et al., 2023). Similarly, combined molecular and agro-morphological approaches have proven effective for characterizing ancient wheat landraces (Gurcan et al., 2017). Although high−throughput SNP based approaches now provide higher genome coverage and resolution, SSR markers remain a practical and cost−effective choice for initial characterization studies, particularly in large−genome species like pea where genome wide SNP genotyping may still be resource intensive for large collections.

Although several SSR−based studies have investigated the genetic diversity of pea, systematic evaluations of a phenotype−guided core collection integrating germplasm from multiple regions remain limited. A comprehensive assessment that links genetic diversity and population structure to ongoing breeding programs is therefore needed. Providing valuable insights into revealing their genetic relationships, classifying ecological types, and preserving core germplasm (Nasiri et al., 2009; Sharma et al., 2022).

To address these issues, after years of effort, we established a temporary germplasm collection of 144 accessions selected for the present study based on their phenotypic and morphological characteristics. We hypothesize that this population harbors untapped genetic diversity. A comprehensive analysis of the genetic background of this population is of critical importance for accurately evaluating its breeding potential, scientifically constructing a core germplasm repository, and guiding the selection of hybrid parents (Loridon et al., 2005; Smýkal et al., 2017). To this end, this study aims to employ multiple pairs of SSR primers to systematically reveal the genetic characteristics of the population from multiple perspectives.

This study aims to systematically evaluate the genetics of 144 pea germplasm accessions using 26 pairs of highly polymorphic SSR primers. Unlike general germplasm collections, our 144 accessions constitute a purpose−built core set that captures both broad geographic diversity and breeding−relevant phenotypic variation, thereby providing a foundation for subsequent hybrid parent selection and genome−wide association studies. The specific objectives include: (1) assess the level of genetic diversity present in this core set; (2) determine the optimal number of genetic clusters (K) and assign accessions to groups; and (3) evaluate the distribution of genetic variation within and between these groups. Through the comprehensive analysis from these multiple perspectives, this study aims to provide an initial characterization of the genetic structure and genetic relationships of the tested germplasm using SSR markers, acknowledging that higher-resolution approaches would be needed for detailed phylogenetic inference.

## 2 Materials and methods

### 2.1 Plant materials

From the initial 601 samples, we conducted a multi-step screening process based on phenotypic traits, geographical origin, growth habit, growth stage, and usage type. A stratified random sampling strategy was adopted. Ultimately, 144 samples were selected as the research materials, accounting for approximately 24% of the original total. These accessions were collected from diverse geographical regions, including China, Australia, Bulgaria, France, the Czech Republic, the United States, Nepal, the former Soviet Union, New Zealand, and the United Kingdom. Among them, 104 accessions were from China.

### 2.2 DNA extraction

For each of the 144 accessions, a single representative plant was selected. When the plants were approximately 14–21 days old, young leaf tissue was collected. Because the young leaves were small, five leaflets were collected from the same plant to obtain sufficient tissue mass for DNA extraction. DNA was extracted from the pooled leaflets of that plant using a modified CTAB method (Dellaporta et al., 1983). Leaf tissue was ground in liquid nitrogen, incubated with extraction buffer (100 mM Tris-HCl pH 8.5, 500 mM EDTA, 1 M NaCl, 10 mM β-mercaptoethanol) and 2% SDS at 65 °C for 1 h, followed by potassium acetate precipitation of proteins and polysaccharides. DNA was precipitated with isopropanol, washed, and purified with a second isopropanol-sodium acetate step. The purity and concentration of the DNA were assessed by 1.0% agarose gel electrophoresis and NanoDrop spectrophotometry. The DNA concentration of all samples was uniformly adjusted to 25 ng/μL and stored at -20 °C for subsequent use.

### 2.3 Polymerase chain reaction amplification

PCR products were separated by polyacrylamide gel electrophoresis (PAGE). Specifically, 6% non−denaturing polyacrylamide gels prepared in 1× TBE buffer were used. Electrophoresis was performed at 120 V for approximately 2.5 hours. After electrophoresis, gels were stained with 0.1% silver nitrate following a standard silver staining protocol (Bassam et al., 1991), and then visualized on a white light box. Fragment sizes were estimated by comparison with a 50 bp DNA ladder (Sangon Biotech (Shanghai) Co., Ltd., China). SSR alleles were scored manually based on the presence or absence of bands at expected fragment sizes for each locus across all accessions. Only clear and reproducible bands were recorded; ambiguous or weak bands were re−amplified and re−run to confirm reproducibility. These SSR markers were originally developed and published in previous studies (Xingbo, 2014), and their polymorphism and reproducibility have been validated in pea germplasm.

The PCR reaction was conducted in a 20 μL volume, consisting of 10 μL of 2× Taq PCR Master Mix, 0.5 μM each of forward and reverse primers, approximately 50 ng of template DNA, and sterile deionized water to adjust the final volume. The PCR amplification program was as follows: initial denaturation at 94 °C for 5 min; followed by 35 cycles of denaturation at 94 °C for 30 s; for each SSR primer pair, a gradient PCR was performed with temperatures ranging from 52 °C to 60 °C to determine the optimal annealing temperature, and extension at 72 °C for 1 min (adjusted based on amplicon length, typically 1 kb/min); with a final extension step at 72 °C for 5 min. Amplification was performed using an ABI Veriti Gradient Thermal Cycler.

### 2.4 Genetic diversity analysis

PowerMarker 3.25 (Liu and Muse, 2005; Razavi et al., 2017) software was employed to analyze the genetic diversity of the 144 pea germplasm resources based on the 26 pairs of SSR primers. Statistical parameters, including the number of observed alleles, effective number of alleles, Shannon’s information index, expected heterozygosity, observed heterozygosity, and polymorphism information content, were calculated to assess marker polymorphism and evaluate the level of genetic diversity among the tested materials.

### 2.5 Population structure and cluster analysis

Population structure analysis was conducted using the Bayesian clustering method with STRUCTURE 2.3.4 (Evanno et al., 2005; Niu et al., 2019) software. The number of assumed populations (K) was set from 1 to 10. This range was chosen because: previous SSR−based studies in pea typically detect 2–6 genetic clusters, with 26 SSR markers, the statistical power to reliably detect very small or highly differentiated subpopulations (K > 10) is limited, and larger K values are more prone to overfitting. In the Markov chain Monte Carlo (MCMC) simulation, a “burn−in” period of 10,000 iterations was set for each independent experimental run, followed by 50,000 effective iterations for subsequent analysis. These parameter values were chosen based on previous SSR−based population structure studies in self−pollinated crops (Kwon et al., 2012; Sharma et al., 2022), The optimal K was determined using the Evanno method (Evanno et al., 2005). To assess whether the MCMC chains had reached convergence, we examined the consistency of the estimated log probability of the data (LnP(K)) across the 20 independent runs for each K value. The standard deviation of LnP(K) among runs was small for all K values (1-10), indicating stable estimation. We further used the online platform CLUMPAK (https://clumpak.evolseq.net/) to align replicate runs and verify that the membership coefficients (Q−values) for K = 2 were highly consistent across runs. These checks confirm that the MCMC chains reached convergence for the primary grouping (K = 2). Accessions with Q ≥ 0.80 were assigned to a genetic group, while those with Q between 0.20 and 0.80 were classified as admixed. This threshold ensures strict assignment of pure lineages while recognizing individuals with shared ancestry. These checks confirm that the MCMC chains reached convergence for the primary grouping (K = 2). The results were subsequently analyzed and visualized using CLUMPAK. Cluster analysis was performed based on Nei’s genetic distance (Nei, 1978), and a phylogenetic tree was constructed using the unweighted pair group method with arithmetic mean (UPGMA) (Gronau and Moran, 2007). Due to the large number of accessions (n = 144), bootstrap resampling was not performed on the full UPGMA tree, as bootstrap support values are known to be unreliable for large trees with highly similar terminal branches (Hillis and Bull, 1993). Consequently, we focus our interpretation on the two major groupings rather than fine-scale branch topology. The tree diagram was visualized using MEGA (Kumar et al., 1994) software to elucidate the genetic relationships and delineate genetic clusters among the germplasm accessions.

### 2.6 Principal component analysis and molecular variance analysis

Using R, the raw SSR genotype data were transformed into an allele dosage matrix employing a binary encoding approach: homozygous genotypes were coded as 1, heterozygous genotypes as 0.5, and missing values were replaced with the mean value of the respective allele. The encoded matrix was subsequently centered and scaled (standardized) prior to PCA. The genetic structure was visualized by plotting the first two principal components, with the proportion of variance explained by each component indicated in the axis labels aiming to visualize the genetic relationships and distribution patterns among germplasm accessions in a two-dimensional space through dimensionality reduction. Molecular variance analysis (AMOVA) was performed using GenALEX 6 (Peakall and Smouse, 2006) software. Based on the population groupings determined from prior cluster analysis, this method quantified and assessed the distribution of genetic variation across different hierarchical levels (between and within populations) and calculated the genetic differentiation coefficients between populations.

## 3 Results

### 3.1 SSR polymorphism analysis of pea germplasm resources

**Figure 1:**
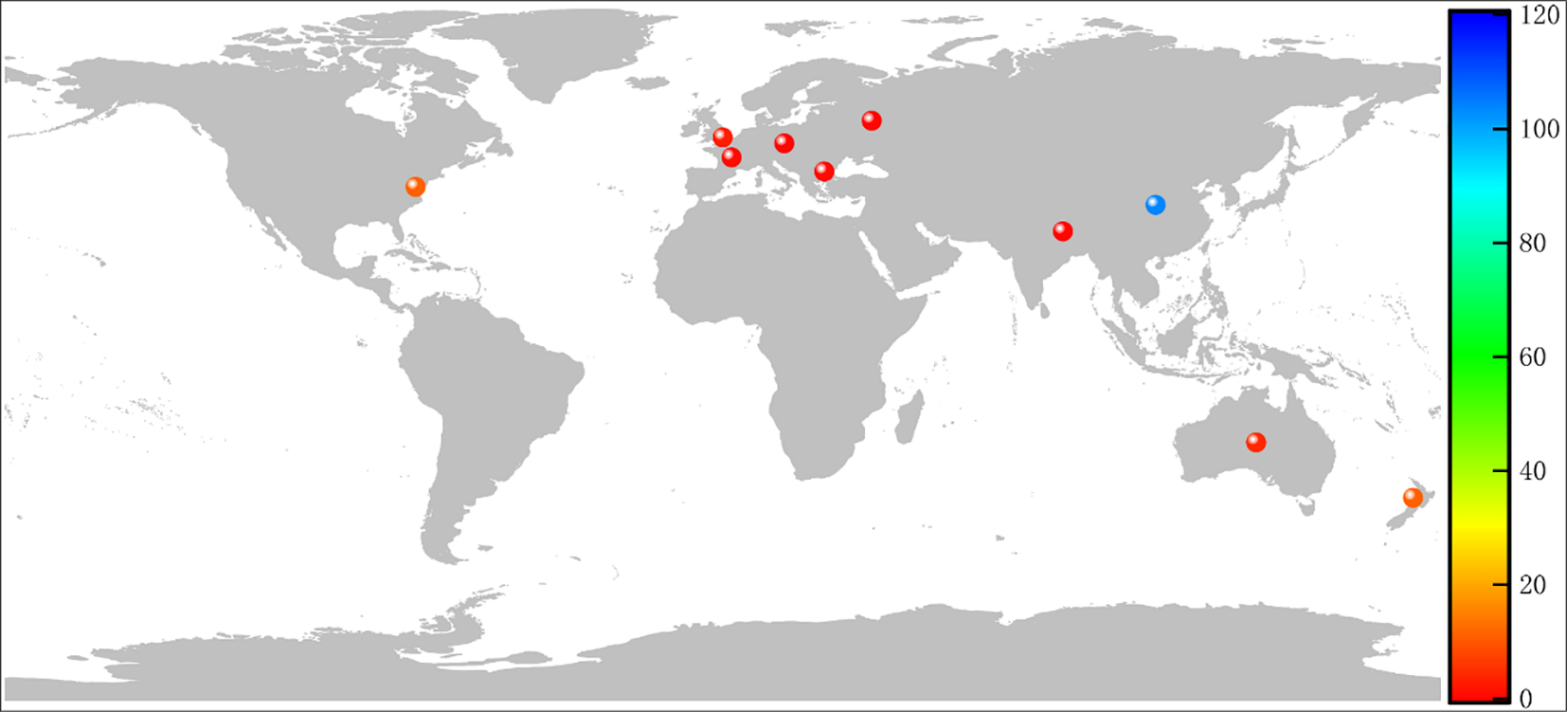
Geographic distribution map of 144 pea germplasm resources collected worldwide. Dot positions indicate geographic origin. Color intensity shifts from blue (more accessions) to red (fewer accessions).

This study conducted SSR analysis on 144 pea germplasm resources from different countries, the majority of the pea germplasm accessions were collected from China. Using DIVA-GIS, all samples were mapped based on geographic location information (**Figure 1**). Detailed information is provided in (**Supplementary Table 1**). A total of 128 alleles were detected across the 26 SSR loci (**Table 1**) (Xuelian, 2013). Different primer pairs were automatically designed from different SSR-containing sequence contigs. Because the pea genome is large and contains many repetitive regions, short primer sequences that happen to fall within conserved flanking regions of different SSR motifs can share high similarity. The average number of observed alleles per locus (Na) was 4.923, ranging from 2 to 10. The average number of effective alleles per locus (Ne) was 2.130, with the highest value (5.219) at locus PSAA497 and the lowest (1.247) at locus 4156. The Shannon’s information index (I) ranged from 0.384 to 1.784, with a mean of 0.895, indicating rich genetic diversity among the tested pea materials. The expected heterozygosity (He) ranged from 0.198 to 0.808, with a mean of 0.468, while the observed heterozygosity (Ho) had a mean of 0.207. The polymorphism information content (PIC) values ranged from 0.1864 to 0.7806, with a mean of 0.4219 (**Table 2**), demonstrating that the markers are suitable for genetic diversity analysis of the tested materials.

**Table 1:** Information of the 26 SSR markers.

Marker	Forward primer sequence (5’→3’)	Reverse primer sequence (5’→3’)
PSAD280	TGGTGCTCGTGATTAATTTCACATA	ACTAAACAACCAACTGCCAAAACTG
PSAD270	CTCATCTGATGCGTTGGATTAG	AGGTTGGATTTGTTGTTTGTTG
PSAD83	CACATGAGCGTGTGTATGGTAA	GGGATAAGAAGAGGGAGCAAAT
PSAC75	CGCTCACCAAATGTAGATGATAA	TCATGCATCAATGAAAGTGATAAA
PSAA497	TTGTGACTGATTTAGAAGTTTCCCAC	TTGATGAGTTGCAATTTCGTTTC
AD134	TTTATTTTTCCATATATTACAGACCCG	ACACCTTTATCTCCCGAAGACTTAG
PSAB23	TCAGCCTTTATCCTCCGAACTA	GAACCCTTGTGCAGAAGCATTA
PB14	GAGTGAGCTTTTTAGCTTGCAGCCT	TGCTTGAGAACAGTGACTCGCA
PSAC58	TCCGCAATTTGGTAACACTG	CGTCCATTTCTTTTATGCTGAG
2200	TGGTTCCTCTGTTGGTCGAG	ACACACACACATACACGGCG
1905	GCACGCACACGAATACAGTC	GACGTGTCGAGTTTGCATGT
4114	ATACACGCATGGCACGATTA	GTACGAGCTTTTGTCACGCA
4156	ACACGCATGCACGATTACAT	GTGTCGTGTACGAGCTTTGC
4629	ACACCATTGCACCATTCTGA	GTGCGTGTGTGTGTGAGTGA
4581	ACACCATTGCACCATTCTGA	GTGCGTGTGTGTTGTGAGTG
5412	CAGAAAAGGAAGCAAGGTGC	GTGAGCAATCTCTCCGGGTA
5540	CAGAAAAGGAAGCAAGGTGC	AGGCAGAGGTTGTGAGCAAT
3494	GCACCGCTCTGACACTCATA	TGAGAGTGGAGTGGCTGAAG
3695	ACACGCATGCACGATTACAT	TTGTCACGCATGTGTATGTGTT
2614	ATGTGTGTGCGTGTGTGTTG	GATTGTTATGTGCTGCGTGG
4043	ACACGCATGCACGATTACAT	CGTGTACGTAGCTTTGCACG
3244	CTTCCCCTCGCAATTTATGA	ATGTGTGTGCGTGTGTGTTG
4816	CGTCATCATTGTTCGTCATTCT	GGTCGTAGGGTGTGTCGTCT
4013	ACACGCATGCACGATTACAT	GTACGAGCTTTTGTCACGCA
3618	GGGAACCCTTTTCTTTTTGC	TGCCATGAGGGAGTCTTAGG
5400	CAGAAAAGGAAGCAAGGTGC	GTGAGCAATCTCTCCGGAAC

**Table 2:** Polymorphism indices of the 26 SSRs.

No.	Marker	Na	Ne	I	Ho	He	PIC
1	PSAD280	2	1.986	0.69	0.917	0.497	0.3733
2	PSAD270	8	4.632	1.784	0.292	0.784	0.7598
3	PSAD83	3	1.511	0.558	0.146	0.338	0.2875
4	PSAC75	8	1.532	0.768	0.028	0.347	0.3278
5	PSAA497	8	5.219	1.765	0.958	0.808	0.7806
6	AD134	5	2.519	1.064	0.021	0.603	0.5219
7	PSAB23	6	1.728	0.903	0	0.421	0.4
8	PB14	5	1.934	0.875	0.021	0.483	0.4225
9	PSAC58	10	2.754	1.481	0.014	0.637	0.6136
10	2200	8	3.483	1.545	0.993	0.713	0.6686
11	1905	5	1.539	0.612	0.417	0.35	0.3022
12	4114	6	1.619	0.682	0	0.382	0.3286
13	4156	4	1.247	0.429	0.132	0.198	0.1895
14	4629	4	1.551	0.661	0.106	0.355	0.32
15	4581	2	1.332	0.415	0.056	0.249	0.2181
16	5412	4	2.17	1.032	0.014	0.539	0.5017
17	5540	4	1.969	0.951	0.16	0.492	0.4581
18	3494	5	1.907	0.906	0.039	0.476	0.4309
19	3695	5	2.232	0.976	0.021	0.552	0.4868
20	2614	4	1.588	0.616	0.119	0.37	0.3121
21	4043	4	2.195	0.948	0	0.544	0.4836
22	3244	3	1.533	0.571	0.045	0.348	0.2946
23	4816	3	1.927	0.692	0.763	0.481	0.3688
24	4013	3	1.257	0.384	0	0.204	0.1864
25	3618	5	2.003	1.016	0.094	0.501	0.4715
26	5400	4	2.013	0.952	0.033	0.503	0.4618
	Mean	4.923	2.13	0.895	0.207	0.468	0.4219

### 3.2 Population structure analysis

**Figure 2:**
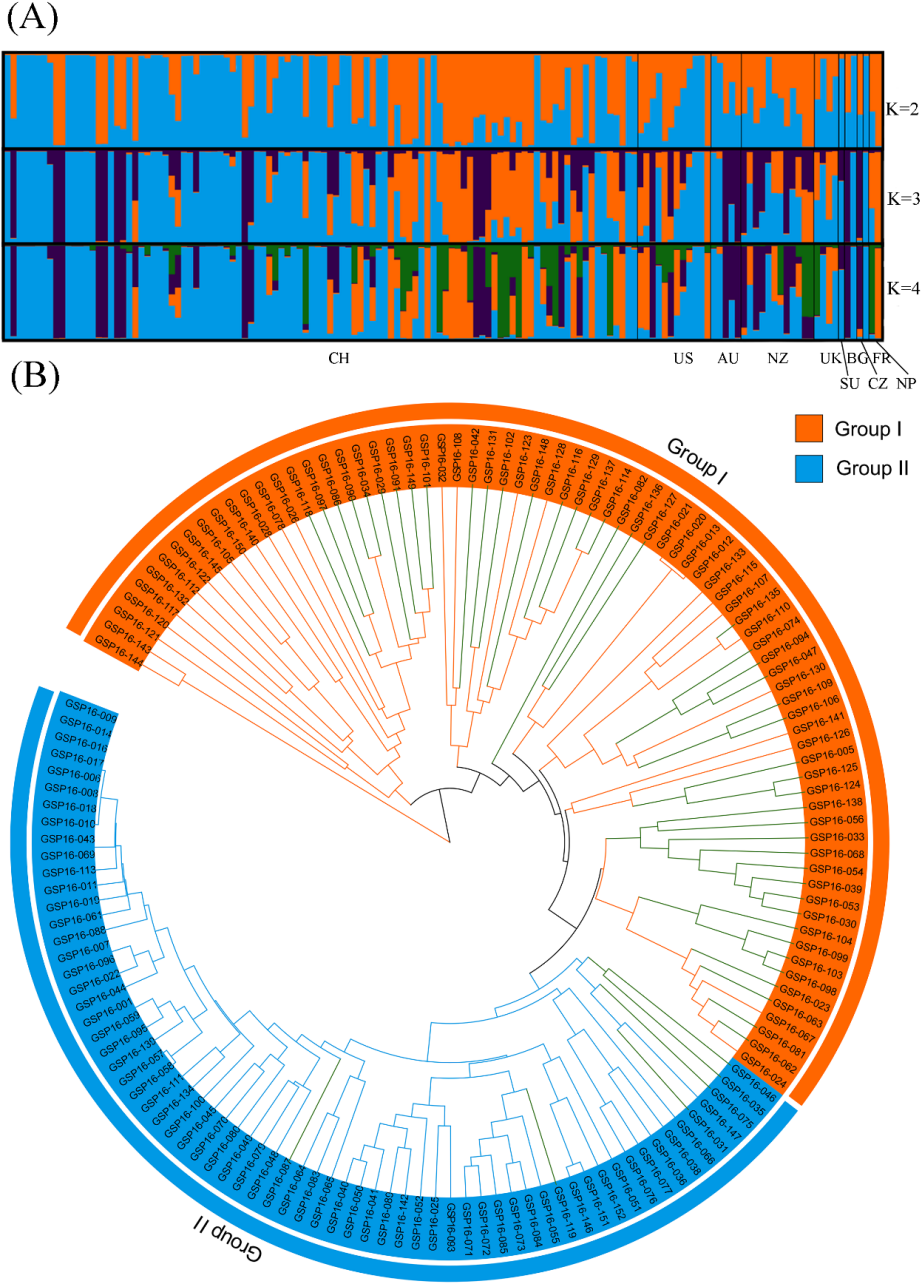
Population genetic structure analysis of 144 pea germplasm resources. **(A)** Population structure based on the Bayesian model. At K = 2, K = 3, and K = 4, the ancestral components (Q-values) of each individual are represented by stacked bar plots, categorized by country (China, United States, Australia, New Zealand, United Kingdom, Soviet Union, Bulgaria, Czech Republic, France, Nepal). Different colors represent distinct ancestral components. **(B)** UPGMA phylogenetic tree based on genetic distance. Orange and blue represent two distinct groups (Group I and Group II), while green branches indicate germplasm identified as having admixed ancestry in the population structure analysis.

Based on Bayesian model-based population structure analysis, germplasm resources from each country exhibited distinct genetic characteristics in the population structure analysis. At K = 2, K = 3, and K = 4, accessions from different countries consistently displayed admixed ancestral components corresponding to diverse geographical origins (**Figure 2A**). When the optimal group number was K = 2, the tested germplasm could be divided into two genetic groups. Based on individual membership coefficient (Q-value) assignments (threshold Q ≥ 0.8), Thirty-two accessions were assigned to Group I, and sixty-one accessions were assigned to Group II. In addition, fifty-one accessions had Q-values between 0.2 and 0.8. The high proportion of admixed individuals suggests substantial shared ancestry or intercrossing between the two major groups.

### 3.3 Cluster analysis

**Figure 3:**
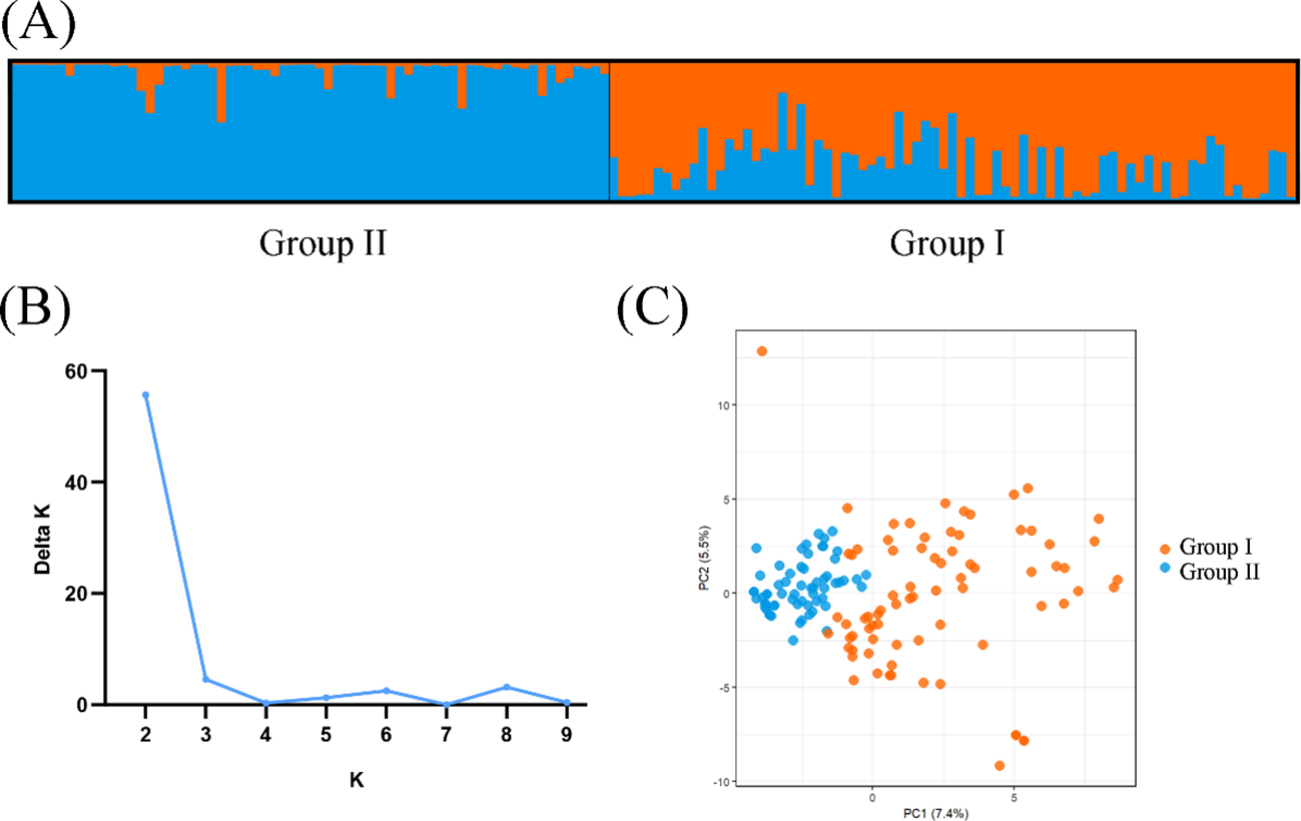
Population genetic structure of tested pea germplasm. **(A)** Stacked bar plot of population structure. Each horizontal bar represents an individual, with its color (orange or blue) indicating the genetic membership of the individual to Group I or Group II. **(B)** Determination of the optimal population number (K-value). The optimal genetic grouping number was determined to be 2 (K = 2). **(C)** Scatter plot of principal component analysis. Points in the plot represent individuals, with colors corresponding to the two main groups (Group I and Group II) defined by Principal Component analysis.

The UPGMA clustering results divided the tested materials into two major groups (Group I and Group II) (**Figure 2B**). Group I consisted of 77 accessions, including all 32 accessions that were assigned to Group I with high confidence (Q ≥ 0.8) in the STRUCTURE analysis, along with 45 accessions classified as having admixed ancestry (Q−values between 0.2 and 0.8). Group II comprised 67 accessions, including all 61 accessions that were assigned to Group II with high confidence (Q ≥ 0.8) in the STRUCTURE analysis, as well as 6 admixed accessions (**Figure 3A**). The internal branches of Group II were extremely short, indicating high genetic similarity and minimal differentiation among its members. In contrast, the internal structure of Group I was more complex, with varying branch lengths, suggesting the possible presence of finer sub-divisions or richer genetic diversity within this group. Determination of the optimal population number (K = 2) further supported this grouping (**Figure 3B**).

### 3.4 Principal component analysis

The results of PCA showed that the first two principal components together explained 12.9% of the total genetic variation (PC1: 7.4%; PC2: 5.5%) (**Figure 3C**), this suggests a multi-dimensional distribution of population genetic structure. The PCA scatter plot visually illustrated the distribution of germplasm in a two-dimensional genetic space. All germplasm accessions were distinctly clustered into two groups. Although partial overlap was observed between the two groups on the PC1-PC2 plane, a clear overall separation trend was evident. The samples of Group II were relatively concentrated in the plot, whereas those of Group I were more widely dispersed., suggesting potentially higher genetic heterogeneity. The PCA visualization is broadly consistent with the STRUCTURE and UPGMA results, they represent three independent analytical approaches model−based assignment (STRUCTURE), distance−based clustering (UPGMA), and low−dimensional visualization (PCA) that all supported the same two−group structure. This convergence strengthens the conclusion that the tested germplasm can be divided into two major genetic groups.

### 3.5 Analysis of molecular variance

AMOVA was performed on the two groups defined by UPGMA clustering (Group I, n = 77; Group II, n = 67). The results of the AMOVA (**Table 3**) indicate that the majority of genetic variation exists within populations. Specifically, variation among populations accounts for only 13% of the total variation, while variation within populations constitutes 87%. The genetic differentiation coefficient among populations is 0.129 and is highly significant (P < 0.001, based on 1,000 permutations). This suggests that although two distinguishable genetic groups exist, the degree of genetic differentiation between them is relatively weak, with rich genetic diversity primarily residing within each population.

**Table 3:** AMOVA based on two genetic groups.

Source	SS	MS	Est. var.	%	FST	P-value	Permutations
Among pops	133.941	133.941	0.892	13%	0.129	0.001	1000
Within pops	1728.028	6.042	6.042	87%			
Total	1861.969		6.935	100%			

## 4 Discussion

### 4.1 Genetic diversity characteristics of the tested pea germplasm resources

A systematic genetic diversity analysis of 144 pea germplasm resources was conducted based on 26 pairs of SSR markers. The results indicate that the tested materials exhibit rich genetic variation. The Na = 4.923 and He = 0.468 obtained in this study are comparable to those reported in previous studies using SSR markers to assess germplasm genetic diversity (Hao et al., 2015; Rana et al., 2017). Our diversity parameters are consistent with previous pea SSR studies: mean He (0.468) is within the reported 0.42–0.52 range, and mean Na (4.923) exceeds the typical 3.2–4.1 range (Kwon et al., 2012; Smýkal et al., 2012), likely due to our broader geographic sampling. We therefore characterize the genetic diversity as moderate to relatively high. It is noteworthy that the Ho = 0.207 was significantly lower than the He = 0.468, suggesting possible inbreeding or selfing within the tested population. This observation aligns with the biological characteristics of peas as a self-pollinating crop (Baranger et al., 2004, Jing et al., 2010). The excess of expected over observed heterozygosity reflects a deficit of heterozygotes within accessions, consistent with the species’ mating system and the predominantly homozygous nature of individual pea plants. This also supports the use of single−plant sampling per accession for population−level analysis, as genetic variation is mostly partitioned among, rather than within accessions.

### 4.2 Population genetic structure and its formation mechanisms

Population structure analysis revealed that the tested germplasm was divided into two main genetic groups at the optimal K = 2, comprising 32 and 61 accessions, respectively. The relatively high proportion (35.4%) of admixed individuals may reflect shared ancestry or potential historical admixture between the two main groups. Germplasm resources from different countries consistently showed admixture of ancestral components at K = 2, K = 3, and K = 4. The observed admixture patterns are largely driven by the diversity among accessions, providing crucial insights for understanding their adaptation mechanisms and unlocking their breeding potential (Brhane and Hammenhag, 2024). UPGMA cluster analysis further validated the results of the population structure analysis, dividing the tested materials into two major groups, Group I and Group II. The extremely short internal branches of Group II indicate high genetic similarity, while Group I exhibited a more complex internal structure with varying branch lengths, suggesting the possible presence of finer sub-divisions within this group (Kwak and Gepts, 2009). This divergence in population genetic structure is may be associated with factors such as effective population size, divergence history, degree of gene flow, and geographic or ecological isolation.

### 4.3 Spatial distribution pattern of genetic variation

PCA showed that the first two principal components together explained only 12.9% of the total genetic variation, indicating that the genetic structure is multidimensional. The plot revealed a tendency toward separation between the two STRUCTURE−defined groups, with Group II samples more concentrated and Group I samples more dispersed. However, substantial overlap was also evident, and no strong clustering claim can be made from the PCA alone. This observation is broadly consistent with the UPGMA clustering, but both methods suffer from the limited resolution of the 26 SSR markers. AMOVA further quantified the distribution of genetic variation, showing that 87% of the variation resided within populations and 13% among populations (Fst = 0.129, P < 0.001). An Fst of 0.129 falls into the range of moderate differentiation. This pattern - most variation within groups - is typical for self−pollinating crops like pea, where limited outcrossing and strong genetic drift within locally adapted populations often preserve diversity within rather than between groups (Hamrick and Godt, 1996). The significant P−value reflects that the among−group component, although modest, exceeds what would be expected under random mating. The genetic differentiation coefficient between populations (Fst = 0.129) reached significant level, confirming significant genetic differentiation between the two groups.

### 4.4 Breeding implications of the research findings

The genetic diversity characteristics and population structure information of pea germplasm resources revealed in this study provide important references for pea genetic improvement and breeding efforts. First, the rich genetic variation observed in the tested materials offers a substantial genetic foundation for selective breeding and hybridization breeding. Fully exploiting this diversity is crucial for achieving future breakthroughs in breeding (Tanksley and McCouch, 1997; McCouch et al., 2013). Second, the population is divided into two main genetic groups, with 35.4% of accessions identified as admixed individuals. Therefore, crossing representative accessions selected from Group I and Group II may generate novel allele combinations that would not be achievable through within-group crosses alone. Nevertheless, these findings provide only a preliminary overview; a more comprehensive dataset with additional SSR markers would enhance the resolution of genetic diversity and population structure analyses. Consequently, we do not claim that inter-group crossing will necessarily lead to improved outcomes; rather, we suggest that the genetic group structure identified here can help guide parent selection in future crossing experiments designed to test this hypothesis (Annicchiarico et al., 2021). Third, the complex genetic structure within Group I suggests that this group may harbor more valuable genetic variations and should be prioritized for further exploration. The findings of this study provide a theoretical foundation and practical direction for broadening the genetic basis of peas, optimizing parental selection, and improving breeding efficiency. However, because the group assignment of individual accessions is based on a limited set of SSR markers, we recommend validating group membership with additional markers or phenotypic data before making large−scale crossing decisions.

### 4.5 Conclusion and prospects

In summary, this study systematically evaluated the genetic diversity and population structure of a working core collection of 144 pea accessions using 26 SSR markers. The main findings are as follows: The SSR markers revealed moderate to relatively high genetic diversity, with mean Na = 4.923, He = 0.468, and PIC = 0.422. Population structure analysis (K = 2) divided the accessions into two major genetic groups (Group I and Group II), with 35.4% of accessions showing admixed ancestry (Q−values between 0.20 and 0.80). AMOVA showed that most of the genetic variation resides within populations (87%), while variation among populations accounts for 13% (Fst = 0.129, P < 0.001). This information can help breeders prioritize accessions for crossing trials. However, empirical hybrid performance tests and phenotypic evaluations are necessary to confirm any actual agronomic advantage or heterosis. While 26 SSR loci cannot capture whole-genome resolution, simulation studies in self-pollinated crops indicate that 20–30 polymorphic SSRs with PIC >0.4 are sufficient to resolve major population subdivisions (K = 2–4) (Urrestarazu et al., 2015). The high congruence among STRUCTURE, UPGMA, and PCA confirms that our marker number is adequate for the main conclusion of two genetic groups. Due to the strong sampling bias toward China (104 out of 144 accessions), the results of population structure analyses should be interpreted with caution regarding geographic patterns. We also acknowledge that future SNP-based genotyping will provide higher resolution for fine-scale structure and GWAS. The results provide a scientific foundation for the conservation, evaluation, and utilization of pea germplasm resources, while also establishing a basis for subsequent molecular marker-assisted breeding, genome-wide association studies, and core germplasm construction (Breseghello and Sorrells, 2006; Korte and Farlow, 2013). Future research could further integrate phenotypic trait data to identify molecular markers associated with important agronomic traits and utilize whole-genome resequencing technology to gain deeper insights into the genetic diversity and evolutionary history of peas.

## Data Availability

The original contributions presented in the study are included in the article/**Supplementary Material**. Further inquiries can be directed to the corresponding authors.
